# Accuracy of diagnosis and evaluation of first ray hypermobility in symptomatic and asymptomatic hallux valgus patients using ultrasonography

**DOI:** 10.1186/s13018-025-05856-3

**Published:** 2025-05-15

**Authors:** Jirawat Saengsin, Pichitchai Atthakomol, Nuttaya Pattamapaspong, Tanawat Vaseenon

**Affiliations:** 1 Department of Orthopaedics, Faculty of Medicine, Chiang Mai University, Chiang Mai, Thailand; 2 Department of Radiology, Faculty of Medicine, Chiang Mai University, Chiang Mai, Thailand

**Keywords:** Ultrasound, First metatarsal, Hypermobile, Hallux valgus, Bunion

## Abstract

**Background:**

Hypermobility of the first ray is related to many foot problems. Existing methods to assess this condition have diverse advantages and disadvantages. The aims of this study were 1) to define the optimal cutoff points for ultrasound evaluation of first ray hypermobility reliant on Klaue device and 2) to evaluate the relationship between the demographic or radiological factors, and hypermobility or forefoot symptoms in patients with hallux valgus.

**Methods:**

Thirty-two hallux valgus patients were enrolled. Patient's demographic and radiographic data were taken. Hypermobility of the first ray was assessed in all patients using both the Klaue device and ultrasound. Ultrasonographic evaluation, including dorsal translation, plantar and medial gapping of the first metatarsal cuneiform joint, was accomplished. Youden's J statistic was calculated for each sensitivity/ specificity pair to determine the optimal cutoff value for the ultrasound measurements to distinguish hypermobile from non-hypermobile group. Continuous outcome data were analyzed using the two-sample t-test. Categorical outcome data were analyzed using Fisher's exact tests. A separate multivariable logistic regression was used to re-evaluate individual variables that were significant on the univariate analysis while controlling for age, sex, and body mass index.

**Results:**

The cutoff points for each ultrasound parameter to diagnose hypermobility of the first ray were determined to be ≥ 1 mm increase in dorsal translation (sensitivity = 96.97%, specificity = 82.61%) or ≥ 0.8 mm increase in medial gapping (sensitivity = 72.73%, specificity = 86.96%) or > 1.1 mm increase in plantar gapping of the first metatarsocuneiform joint (sensitivity = 81.82%, specificity = 91.30%). The increase in width of the foot and the increase in hallux valgus angle were correlated with hypermobility of the first ray, while the increase in hallux valgus angle and the first–second intermetatarsal angle were correlated with symptoms in hallux valgus patients.

**Conclusions:**

The ultrasound technique measuring three parameters of the first ray motion provides acceptable accuracy for the first ray mobility assessment. Width of the feet and hallux valgus angle were associated with hypermobility of the first ray while an increase in the hallux valgus angle and the first–second intermetatarsal angle were associated with symptoms among hallux valgus patients.

## Introduction

The first ray of the foot incorporates the first metatarsal bone, the first metatarsocuneiform joint, and the medial cuneiform bone. Because they function as a weight-bearing structure, it necessary to provide enough stability to withstand high loads and force during the gait cycle. The stability of the first ray depends on the bony configuration, articular congruity, and capsuloligamentous tissue, especially the dorsal, plantar, and interosseous ligaments between the metatarsal and the cuneiform bone [1–4]. Together these structures stabilize motion in three planes (axial, coronal, and sagittal).

Hypermobility of the first ray resulting in foot symptoms due to sagittal plane instability was first proposed in 1928 by Morton [5]. When instability occurs, the load will be transmitted to the lesser metatarsals, producing symptoms such as second metatarsophalangeal joint synovitis, transfer metatarsalgia, and stress fracture [6–8]. As the first ray is capable of multiplanar motion, the classic description of hypermobility of the first ray, which is defined as an increase only in the sagittal plane of motion, may be inappropriate for defining hypermobility.

Several methods to assess and diagnose first ray hypermobility are currently available. The first method is based on clinical assessment in which the first metatarsal head is translated in a dorsal-plantar direction until a soft endpoint is reached and indirectly defines first ray hypermobility as the excess motion of first metatarsal bone relative to the second metatarsal bone is observed [5]. However, this technique is a subjective assessment resulting in poor interobserver and intraobserver reliability. Despite some modifications, for example, a ruler to quantify mobility, the reliability and validity were still limited when compared with device measurement [9, 10].

**Fig. 1:**
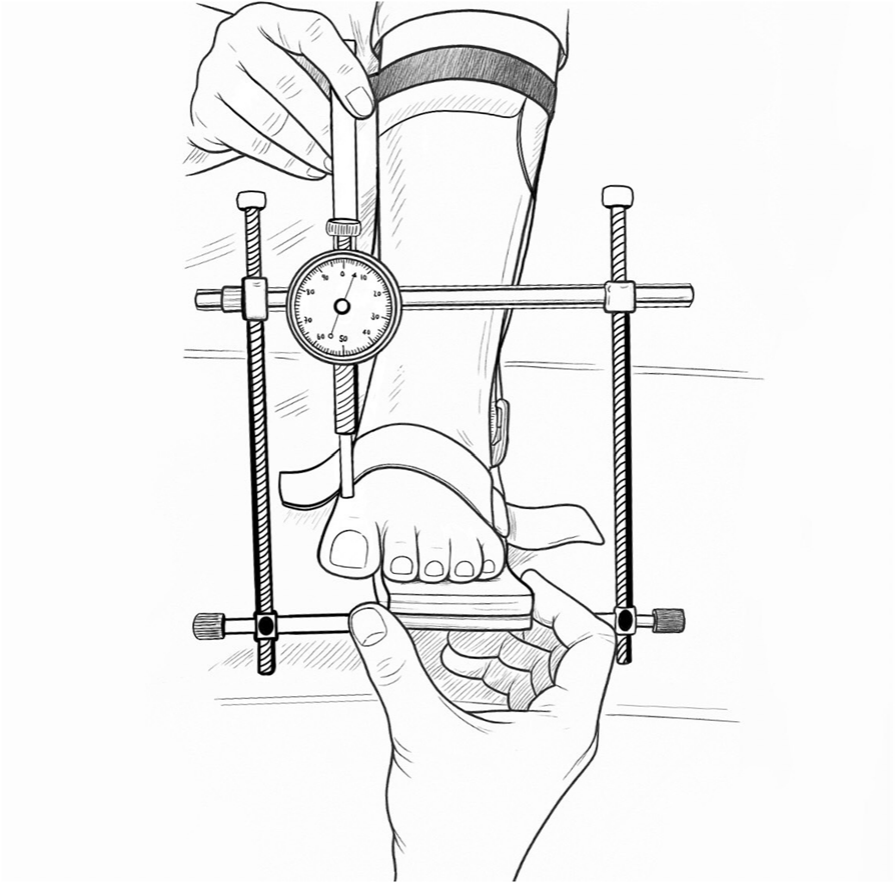
The illustration of Klaue device

One potential method to more accurately quantify first ray mobility is the"Klaue device". This device measures the magnitude of first ray hypermobility in millimeters of dorsal translation of the first ray relative to the stabilized second metatarsal bone [11]. Studies indicate that this device provides reliable and replicable quantitative values for both normal and hypermobility of the first ray in hallux valgus patients [12]. Despite quantitatively measuring overall metatarsal translation, this device assesses only the vertical plane of motion. (Fig. 1: Klaue device).

In order to improve assessment, Glasoe et al. developed another device to evaluate first ray mobility [13]. This device controls the force applied to the first ray and has shown to be reliable and reproducible measurements comparable to the Klaue device. However, due to the complexity and larger size of the Glasoe device, its use is impractical for routine clinical evaluations, and the Klaue device is still employed as the preferable tool.

Ultrasonography is another alternative method for evaluating joint instability. Ultrasound is convenient because the equipment is easily available and accessible. Besides, advances in ultrasound technology, including improved accuracy of images as well as the ability to provide dynamic, multiple planes, and real-time pictures, have increased its efficacy for evaluating joint stability [14–24].

There are several studies on the ultrasound-assisted diagnosis of the musculoskeletal system, including screening and diagnosis of fractures or ligamentous injuries [15–19, 25, 26]. Similarly, studies have highlighted the use of ultrasound-assisted procedures and surgeries, including in the field of orthopedics, e.g., ultrasound assistance in intramedullary nailing of femoral shaft fractures [27, 28], ultrasound assistance in the reduction and treatment of distal radius fractures [29, 30], and the use of ultrasound to assist minimal invasive surgery in femoral shaft fractures [20].

To the best of authors'knowledge, there is currently no consensus on the best technique for evaluating the hypermobility of the first ray. The Klaue device is generally considered to be the most reliable and measurable method despite its limitations. Ultrasound, besides many advantages, can be used to evaluate first ray hypermobility. However, there have been no studies specifically investigating the accuracy of ultrasound evaluation of hypermobility of the first ray. The aims of this study were 1) to define the optimal cutoff points for ultrasound evaluation of first ray hypermobility reliant on baseline Klaue device measurement, and 2) to compare the baseline demographic and radiographic information between hypermobile and non-hypermobile patients as well as symptomatic and asymptomatic patients.

## Methods

This study was conducted at Maharaj Nakorn Chiang Mai Hospital between September 2016 and September 2017 with ethical approval from the Research Ethics Committee Faculty of Medicine, Chiang Mai University. Voluntary informed consent was obtained from the subjects before performing any test. After consent obtainment, all hallux valgus patients identified at the Outpatient Clinic were invited to participate in the study. The participants with any history of forefoot or midfoot trauma or other foot surgery were excluded. There were 32 patients (64 feet) who met our criteria. Patient's demographic data were taken, including sex, age, body mass index (BMI), and Beighton score. Patients with clinical symptoms such as bunion pain, transfer metatarsalgia, plantar callosity at second and/or third metatarsal head were also recorded. All patients underwent a weight-bearing radiographic study of their feet in the form of anteroposterior and lateral views to measure the first metatarsophalangeal joint angle and the first–second intermetatarsal angle.

Patient variables were analyzed. Descriptive data were demonstrated with frequencies and percentages for categorical variables and with means and SD for continuous variables. The comparisons were made between the hypermobile and non-hypermobile groups and between the symptomatic and asymptomatic groups. Continuous outcome data were analyzed using the two-sample t-test. Categorical outcome data were analyzed using Fisher's exact tests. A separate multivariable logistic regression was used to re-evaluate individual variables that were significant on the univariate analysis while controlling for age, sex, and BMI.

The degree of hypermobility of the first ray was evaluated using both a Klaue device and ultrasound. The orthopedic doctors responsible for evaluation using the Klaue device, and the ultrasound did not communicate their findings with each other in order to minimize the bias. Ultrasound evaluations for 32 patients were performed independently by two orthopedic doctors whose experience in the field of musculoskeletal sonography was more than two years. Inter-rater and intra-rater reliability were assessed for ultrasound measurement using intraclass correlation coefficients derived from a 2-way mixed-effect model analysis of variance for absolute agreement. This model was used because the two raters were not randomly assigned, and both raters evaluated first ray mobility using ultrasound in the same patients. Interpretation of the ICC values was carried out according to the guidelines proposed by Shrout: 0.41–0.60 fair, 0.61–0.80 moderate, 0.81–1.00 substantial, or excellent agreement [31].

The Klaue device was constructed according to the original paper by Klaue et al. [11]. To evaluate the hypermobility of the first ray with the Klaue device, each foot was positioned in the device with a neutral ankle position. The feet were preconditioned by applying a manual force across the first tarsometatarsal joint in the dorsal plantar and medial lateral direction a total of 15 times over a period of 5 min. Subsequently, the force was applied under the first metatarsal head in the plantar to the dorsal direction to cause movement in the first metatarsocuneiform joint. When the metatarsal head reached the soft tissue endpoint, a micrometer was used to measure the translation distance of the first metatarsal head relative to the second metatarsal head. It has been reported in the literature that using a Klaue device, a healthy adult has approximately 5.3 ± 1.4 mm of metatarsocuneiform joint mobility, while patients with hallux valgus have mobility greater than or equal to 9.3 ± 1.9 mm [11]. In our study, hypermobility of the first ray was defined as the translation of more than 9.3 mm in subjects evaluated with a Klaue device.

**Fig. 2:**
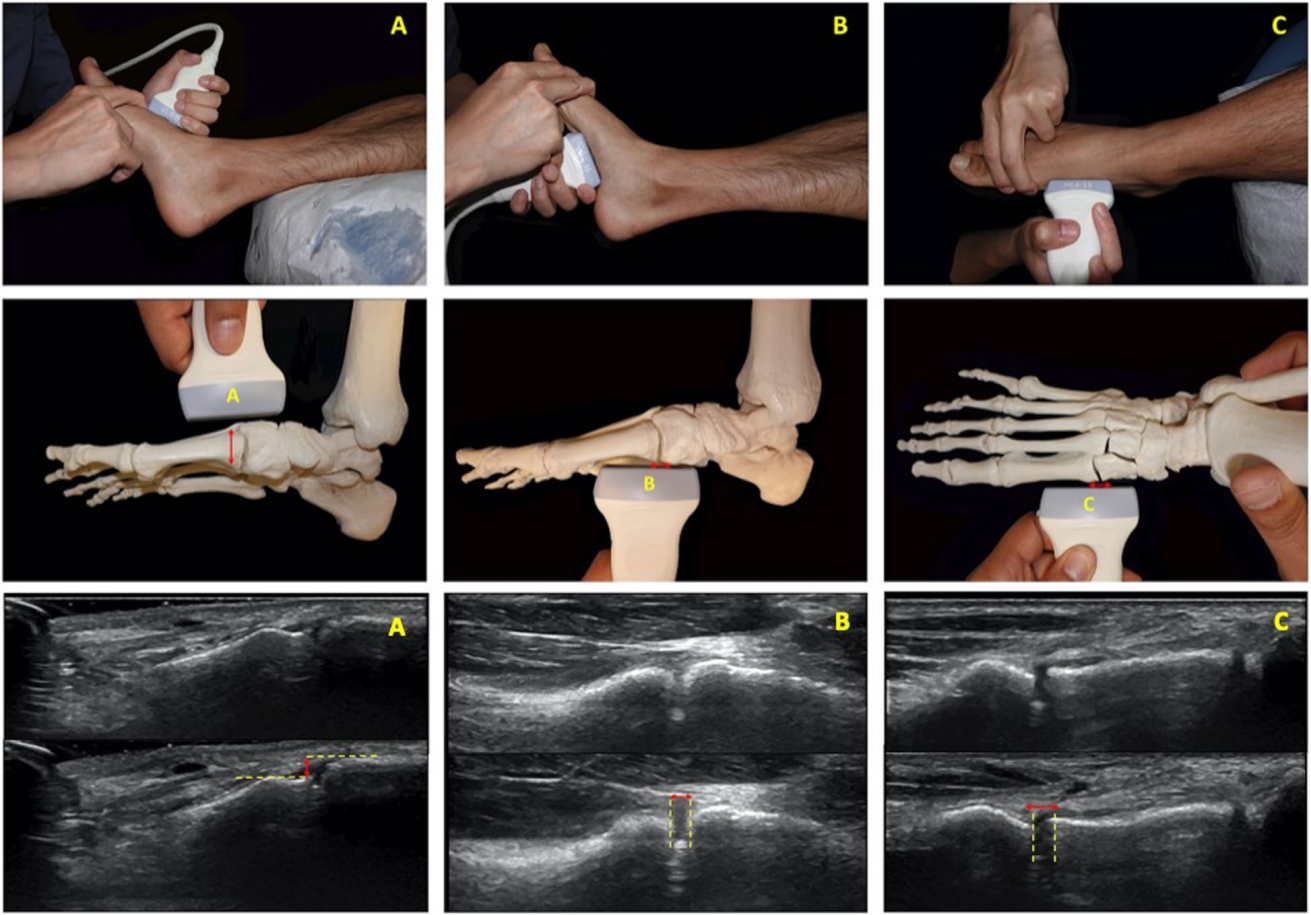
Ultrasound probe orientation and measurement. A probe placed perpendicular to the dorsum of the first metatarsocuneiform joint measures dorsal translation, representing vertical mobility (**A**). A probe placed perpendicular to the plantar side of the first metatarsocuneiform joint measures plantar gapping, representing mobility under weight-bearing (**B**). A probe placed perpendicular to the medial side of the first metatarsocuneiform joint measures medial gapping, representing horizontal mobility (**C**). The techniques of ultrasound measurements before and after sufficient force were applied to reach the soft tissue endpoints and no further bone movements. The distances before and after the application of force were recorded for dorsal translation (**A**), plantar gapping (**B**), and medial gapping (**C**)

Using ultrasound, hypermobility of the first ray was determined by measuring the plantar gap, the medial gap, and dorsal translation at the first metatarsocuneiform joint. These parameters were recorded as a delta values between the distance measured before force was applied and after force applied to the point that the soft tissue endpoint was reached, and no further movement was observed at the joint or until the patient felt discomfort. For these measurements, the ultrasound probe was placed on the area and perpendicular to the first metatarsocuneiform joint in each plane of measurement. To assess dorsal translation, the ultrasound probe was placed at the dorsal aspect perpendicular to the joint. This parameter was measured at the ultrasound-identified bone edge signal of the first metatarsal base, and the medial cuneiform at the level of the joint both before and after the force was applied to translate the first metatarsal dorsally. To assess plantar gapping, the ultrasound probe was placed at the plantar aspect perpendicular to the joint. This parameter was measured at the ultrasound-identified bone edge of the first metatarsal base, and the medial cuneiform at the level of the joint from the plantar aspect before and after the force was applied to translate the first metatarsal dorsally. To assess medial gapping, the ultrasound probe was placed at the medial aspect perpendicular to the joint. This parameter was measured at the ultrasound-identified bone edge of the first metatarsal base, and the medial cuneiform at the level of the joint from the medial aspect before and after the force was applied to translate the first metatarsal laterally (Fig. 2).

The association between the first ray translation values measured with the Klaue device and the ultrasound values were analyzed using linear regression. The estimated coefficient, 95% confident interval, and *P* value were reported.

A receiver operating characteristic (ROC) curve analysis was performed using the Klaue device value as a standard reference. A translation of more than 9.3 mm in subjects evaluated with a Klaue device is defined as hypermobility. In a ROC curve, the true positive rate (sensitivity) is plotted as a function of the false-positive rate (100 – specificity) for different cutoff points. Each point on the ROC curve represents a sensitivity/specificity pair corresponding to a particular decision threshold. Youden's J statistic was calculated for each sensitivity/specificity pair (J = sensitivity + specificity – 1) to determine the optimal cutoff value (in millimeters) for the ultrasonographic values to distinguish hypermobile from non-hypermobile group. The area under the ROC curve measures the accuracy of the diagnostic test. An area of 1.0 represents a perfect test, and an area of 0.5 represents a worthless test.

In this study, we used an ultrasound system with HFL38x transducer, 13–6 MHz 38-mm linear broadband array providing scan depths up to 6 cm, which is sufficient to evaluate the musculoskeletal condition. All statistical testing was done by using Stata 14.2 (StataCorp LP, College Station, TX). The significant level was defined as a *P* value < 0.05.

## Results

A summary of the baseline characteristics is displayed in Table 1. Clinical symptoms in all subjects were recorded. Among 32 hallux valgus patients, there were seventeen patients who had symptoms on both feet. Twelve patients had symptoms on one foot, and three patients did not have any symptoms. The three most common chief complaints of the patients were bunion pain, transfer metatarsalgia, and plantar callosities at the second and/or third metatarsal head.

**Table 1:** Baseline characteristics of the study group

Parameters	Mean	Standard deviation (SD)
		/Percentage (%)
Age (years)	51.07	16.44
Gender		
Male (N)	7	21.87%
Female (N)	25	78.12%
BMI (kg/m^2^)	22.69	2.88
Length of feet (mm)	23.38	1.36
Width of feet (mm)	9.74	0.75
Length of first metatarsal bone (mm)	62.83	4.74
Length of second metatarsal bone (mm)	73.74	5.8
HV angle (degrees)	30.03	9.83
First and second IM angle (degrees)	14.86	4.28

When assessing the mobility of the first ray using the Klaue device, eight hallux valgus patients did not have first ray hypermobility. Ten patients had unilateral first ray hypermobility, and fourteen patients had bilateral first ray hypermobility.

**Fig. 3:**
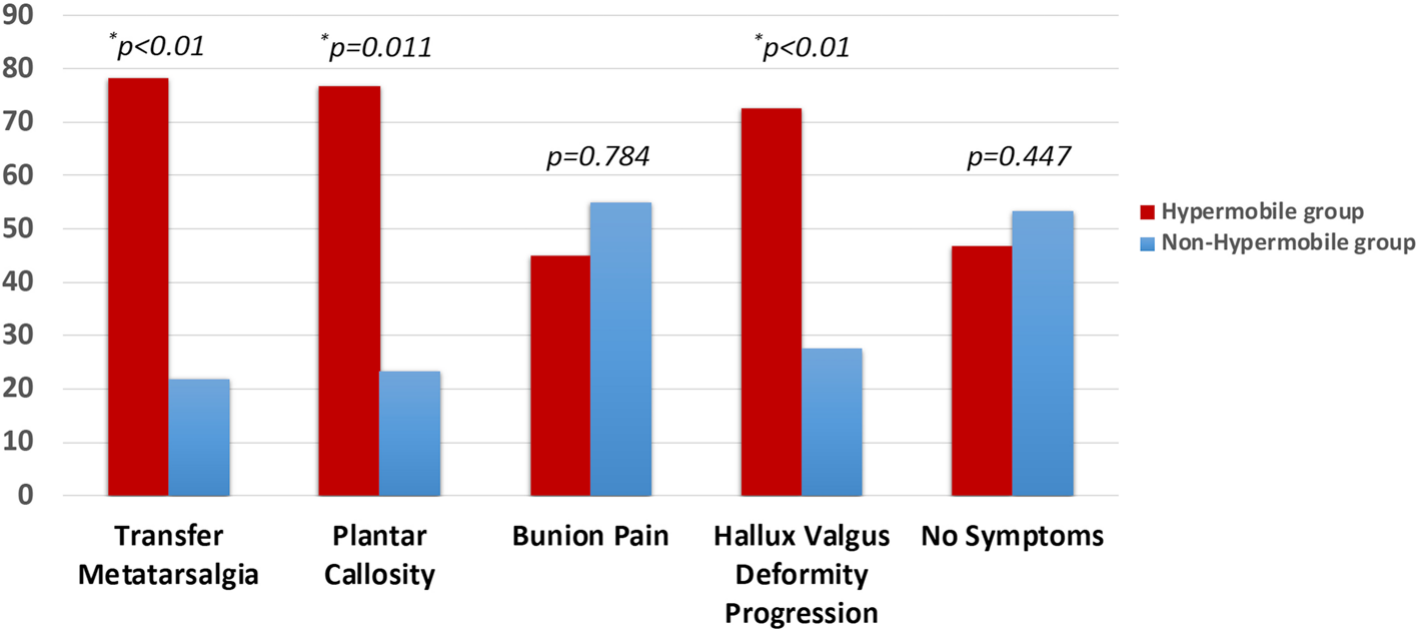
Clinical symptoms in hallux valgus patients with and without hypermobile first ray (%)

Patients with hypermobility of the first ray developed more transfer metatarsalgia (*P* < 0.01), plantar callosities (*P* = 0.011), and deformity progression (*P* < 0.01) when compared with non-hypermobile group (Fig. 3).

We divided patients by mobility status besides symptom status based on Klue device measurement. The two-sample t-test was used to compare these groups. Width of the feet and hallux valgus angle were found to be significantly different between the normal and hypermobile groups (*P* = 0.037, *P* = 0.046). A comparison between the symptomatic group and asymptomatic group found that the hallux valgus angle and the first–second intermetatarsal angle were both significantly higher in the symptomatic group (*P* = 0.004, *P* = 0.028) (Table 2).

**Table 2:** Baseline characteristics comparing hypermobile and non-hypermobile patients as well as symptomatic and asymptomatic patients

Parameter	Mobility status			Symptom status		
	**Hypermobile**	**Non-hypermobile**		**Symptomatic**	**Asymptomatic**	
	(*N* = 24)	(*N* = 8)	*P* value	(*N* = 29)	(*N* = 3)	*P* value
Age, years, mean (SD)	54.5 ± 15.8	46.2 ± 16.5	0.064	51.4 ± 15.9	50.3 ± 18.5	0.83
Sex, male/female, (%)	18.2/81.8	17.4/82.6	1.00	14.6/85.4	26.7/73.3	0.43
BMI (kg/m^2^)	22.7 ± 2.8	22.7 ± 3.1	0.98	22.9 ± 2.4	22.2 ± 3.9	0.47
Beighton score, < 3/≥ 3, (%)	0.7 ± 1	1.7 ± 2.6	0.053	1.3 ± 2.1	0.6 ± 0.98	0.43
Length of feet (mm)	23.3 ± 1.5	23.5 ± 1.1	0.73	23.4 ± 1.4	23.4 ± 1.3	0.97
Width of feet (mm)	9.9 ± 0.7	9.5 ± 0.7	0.037*	9.8 ± 0.8	9.7 ± 0.8	0.79
First metatarsal length (mm)	62.3 ± 4.6	63.5 ± 4.9	0.35	62.4 ± 4.6	63.9 ± 5	0.31
Second metatarsal length (mm)	73.1 ± 6	74.7 ± 5.5	0.33	73.2 ± 5.8	75.3 ± 5.9	0.2
HV angle (degrees)	32.2 ± 9.8	26.9 ± 9.2	0.046*	32.3 ± 8.3	23.9 ± 11.2	0.004*
1–2 IM angle (degrees)	14.9 ± 3.4	14.9 ± 5.4	0.98	15.6 ± 4.6	12.8 ± 2.5	0.028*

Hypermobility = Positive Klaue device > 9.3 mm translation

^*^Statistically significant (*P* < 0.05)

A separate multivariable logistic regression was used to re-evaluate individual variables that were significant on the univariate analysis while controlling for age, sex, and BMI. The hallux valgus angle and the first–second intermetatarsal angle were still found significantly higher in the symptomatic group (Table 3).

**Table 3:** Risk for hypermobility and symptoms in hallux valgus patients: Results of multivariate logistic regression controlling for age, sex and BMI

	**Hypermobility**			
	**Coefficient**	**Standard Error**	**95% CI**	***P* Value**
Width of feet (mm)	0.784	0.491	−0.180, 1.748	0.11
HV angle (degrees)	0.052	0.031	−0.009, 0.113	0.095
	**Symptoms**			
	**Coefficient**	**Standard Error**	**95% CI**	***P* Value**
HV angle (degrees)	0.107	0.039	0.030, 0.183	0.006*
1–2 IM angle (degrees)	0.255	0.127	0.007, 0.504	0.044*

Hypermobility = Positive Klaue device > 9.3 mm translation

^*^Statistically significant (*p* < 0.05)

Three sonographic parameters were developed to evaluate first ray hypermobility. The interclass correlation coefficient showed excellent agreement for both inter-rater (Dorsal translation, 0.87, 95%CI: 0.80–0.92; plantar gapping, 0.88, 95%CI: 0.80–0.93; medial gapping, 0.85, 95%CI: 0.76–0.90) and intra-rater agreement (Dorsal translation, 0.91, 95%CI: 0.86–0.95; plantar gapping, 0.91, 95%CI: 0.85–0.95; medial gapping, 0.87, 95%CI: 0.79–0.92).

The optimal cutoff points were chosen based on the highest area under the Receiver Operating Characteristic (ROC) curve. The cutoff points for the ultrasound parameters used to diagnose hypermobility of the first ray were set at an increase in dorsal translation of ≥ 1 mm, an increase in gapping of ≥ 1.1 mm in plantar gapping, and an increase in medial gapping of the first metatarsocuneiform joint of ≥ 0.8 mm. When we combined these three parameters to give a diagnosis of hypermobility of the first ray, we found improvement in the accuracy of diagnosing hypermobility of the first ray (Table 4).

**Table 4:** Sonographic cut-off points at the first metatarsocuneiform joint for diagnosis of hypermobility of the first ray

Ultrasound parameters	Sensitivity	Specificity	PPV	NPV	Area under ROC curve
(1) Dorsal translation (Cut-off point ≥ 1 mm)	96.97%	82.61%	88.89%	95.00%	0.89*
(2) Plantar gapping (Cut-off point ≥ 1.1 mm)	81.82%	91.30%	93.10%	77.78%	0.86*
(3) Medial gapping (Cut-off point ≥ 0.8 mm)	72.73%	86.96%	70.45%	83.33%	0.68*
(4) Combined (1) (2) (3)	78.79%	95.65%	96.29%	75.86%	0.87*

Using the sonographic measurements, we evaluated the relationship between first ray translation values measured with the Klaue device and these ultrasound parameters using Linear regression analysis. A significant association was observed. We also compared sonographic measurements between a group of patients with and without symptoms. However, the result found no significant difference between these groups (Table 5).

**Table 5:** Sonographic mobility assessment in association with first ray translation values measured with Klaue device and symptom status in hallux valgus patients

Parameters	Symptom status			First ray translation measured		
	**Symptomatic**	**Asymptomatic**		**with Klaue device**		
	**(Mean ± SD)**	**(Mean ± SD)**		**Coefficients**	**95% CI**	***P* value**
	**(*N* = 46)**	**(*N* = 18)**	***P* value**			
**-** Dorsal translation (mm)	1.1 ± 0.62	1.17 ± 0.55	0.69	44.91	34.12, 55.70	< 0.001*
- Plantar gapping (mm)	1.09 ± 0.47	1.16 ± 0.79	0.70	37.50	23.71, 51.28	< 0.001*
- Medial gapping (mm)	0.84 ± 0.47	0.85 ± 0.63	0.98	33.98	17.24, 50.72	< 0.001*

^*^Statistically significant (*P* < 0.05)

## Discussion

First ray hypermobility contributes significantly to the pathology of forefoot problems, especially hallux valgus. However, the lack of a precise definition and evaluation method for this condition has been a continuing obstacle.

In our study, we included many factors in order to find an association with the first ray hypermobility. We found that the width of the feet was significantly different in the normal mobility and the hypermobility groups with univariate analysis. Increased foot width could be the result of joint laxity in the horizontal plane causing wider spreading of the metatarsal bones or medial deviation of the first metatarsal. However, after controlling for age, gender, and BMI, the *P* value did not reach statistical significance.

The radiographic parameters measured in the weight-bearing films failed to show any significant difference between the normal mobility and hypermobility groups. This result is similar to previous reports in the literature that found no correlation between shortness of the first metatarsal or hypertrophy of the second metatarsal and hypermobility of the first ray [32]. In our study, we also found a significant difference only in the hallux valgus angle between the normal mobility and hypermobility groups with univariate analysis. However, after controlling for age, sex, and BMI, the hallux valgus angle did not reach statistical significance.

Previous studies have reported similar results showing a significant increase in dorsal mobility of the first ray in patients with abnormal hallux valgus angle versus those with a normal angle, and a marginal correlation (*r* = 0.51) between the dorsal mobility of the first ray and the first–second intermetatarsal angle [33]. Furthermore, Dietze et al. also showed that the increase in the first–second intermetatarsal angle was significantly correlating with maximum dorsiflexion distance (*r* = 0.817, *P* = 0.013) and maximum dorsiflexion angle of the first ray (*r* = 0.824, *P* = 0.012) when assessed with radiokinematic gait analysis in hallux valgus patients [34].

When comparing the symptomatic and asymptomatic groups, the hallux valgus angle and the first–second intermetatarsal angle were found to be significantly higher in the symptomatic group even after adjusting for age, gender, and BMI (*P* = 0.006, *P* = 0.044) which is in line with prior study [35]. Besides, we found that the number of subjects who experience symptoms including transfer metatarsalgia, plantar callosity, and hallux valgus deformity progression was statistically significantly higher among the hypermobile group when compare with non-hypermobile group. This finding may help to support the theory proposed in 1928 by Morton about the first ray hypermobility causing various forefoot problems [5]. Recently, there is a well-established concept to analyze load transmission in the foot using pedobarographic analysis. Dietze et al. performed an experiment in hallux valgus patients with clinically unstable of the first tarsometatarsal joint. They found that the increased first tarsometatarsal joint mobility resulted in a positive correlation with maximum force under the second (*r* = 0.805, *P* = 0.016), the third (*r* = 0.764, *P* = 0.027) and the fourth metatarsal bone (*r* = 0.807, *P* = 0.015). Their findings help to emphasize the theory about the first ray hypermobility, causing load transmission [34].

However, controversy still exists whether the hypermobility of the first ray, resulting in load transmission, could potentially be a significant cause of pathology, especially in hallux valgus patients as there are several surrounding structures including capsule, tendons, muscles, ligament and plantar aponeurosis which could also play an important roles on both static and dynamic stability of the medial column of the foot. The study from Dullaert et al. showed that the weight-bearing significantly increased Meary's angle and decreased first tarsometatarsal joint subluxation (*P* < 0.01). They also found that the pulling of peroneus longus tendon improves first metatarsal subluxation by significant decrease intermetatarsal angle (*P* < 0.01) and increase Meary's angle (*P* = 0.052). However, the effect of pulling also increases the first metatarsal rotation (*P* < 0.01) [36]. Their findings reaffirmed that the dynamic and static condition of the first ray also provides different stability at the joint, which could result in different values when assessed for hypermobility.

Although hypermobility of the first ray has been frequently mentioned, the debate has continued regarding how to accurately measure and define the pathologic mobility of the first ray. A review of the literature shows that many studies have reported on the prevalence and cutoff points of first ray hypermobility in patients, but each of those studies used their own definitions and experience without clearly delineating a pathologic cutoff point. For example, subjectively define hypermobility from physical examination or a range of motion exceeding the 95 th percentile for the normal population [9].

Our study used a new technique, ultrasound assessment, for first ray hypermobility evaluation, a method that provides for more objective measurement. A review of the literature did not find studies using ultrasound as a tool to diagnose hypermobility or established a clear-cutoff point for the condition. In this study, we attempted to demonstrate that sonography, which is now well-developed, can be used as an alternative method of hypermobility evaluation. Moreover, we tried to assess more planes of motion of the first metatarsocuneiform joint using three sonographic parameters and provide mobility values with quantitative information. The dorsal translation is representative of the vertical mobility of the joint, medial gapping indicates the horizontal plane mobility of the joint, and plantar gapping is a representation of the vertical mobility with force applied from the plantar side which is comparable to weight-bearing status.

Recently, several studies are demonstrating advanced methods of the first ray mobility assessment, such as a 3-D computer tomography imaging, weight-bearing CT scan, radiokinematic, and pedobarographic analysis [34, 36–39]. However, these techniques are costly and not easily available. In addition, it also causes significant radiation exposure to patients.

The ultrasound hypermobility test seemed repeatable, even though we did not control for the forces applied. Interestingly, the values provide with applied forces until it reached soft endpoint by different operators were quite similar. The inter- and intraobserver agreement coefficients of the ultrasound measurements were all excellent.

We also compared the degree of hypermobility between symptomatic and asymptomatic hallux valgus patients. However, our statistical analysis failed to show any significant difference between the symptomatic and asymptomatic groups during the ultrasound mobility assessment.

Our study also has many strengths and potential clinical applications. It aimed to standardize a cheap non-ionizing radiation diagnostic method that is readily available in multiple settings such as in less developed countries, in the out-patient clinic, or sports field, at the point of care [40]. The information provided by this ultrasound test is quantitative and measurable data, which seems repeatable and reproducible. Besides, an ultrasound is now available in a portable model that can be carried and used to assess the patients anywhere.

This reliable technique of ultrasound assessment can shed light on the mobility status of the first ray and guide surgeons to choose appropriate surgical procedure for hallux valgus. Nevertheless, apart from hypermobility of the first ray, multiple factors such as the degree of hallux valgus deformity, the preoperative sesamoid position, the shape and posture of the foot; and the arthritic condition of the metatarsophalangeal joint influences the surgeon's decision while selecting a procedure for hallux valgus correction [41–45].

Our study had some limitations. Firstly, we did not resolve the issue of the level of force that should be used to translate the first ray. We were concerned that the technique should be appropriate for practical application in routine practice as in the previous literature with the method of more precise force applied but not feasible [13]. We used the maximum force to reach the soft tissue endpoint rather than a force measurement device as those devices are not commonly available in out-patient clinics. We believe a practical approach is to apply an increasing level of force until either no further movement of the first ray is detectable by ultrasound or until the patient starts to feel discomfort, whichever comes first. Secondly, we did not assess the axial motion of the first–second metatarsocuneiform joint, one of the motion planes. This could be an interesting topic for future research. Thirdly, we did not explore the values of hypermobility seen in healthy feet when assessed with ultrasound. Finally, our measurement technique involved an operator learning curve. Sonography is a skill that includes a learning process; operators need time to practice. In the hands of an experienced operator, sonographic images can provide useful information without radiation or any other contraindication.

## Conclusion

According to our analysis, these three sonographic parameters have acceptable power to identify hypermobility. In conclusion, the assessment of hypermobility of the first ray is possible using ultrasound. Hypermobility of the first ray should be suspected if there is ≥ a 1 mm increase in dorsal translation, ≥ 0.8 mm increase in medial gapping, and ≥ 1.1 mm increase in plantar gapping of the first metatarsocuneiform joint. Our cutoff point ultrasound parameters reliant on baseline Klaue device measurement. With this technique, it offers physician an alternative way of hypermobility evaluation with a feasible and measurable method.

Further investigation is required to evaluate the ultrasonographic cutoff points to see how this test is associated with the surgical outcomes after the procedure for first ray hypermobility in hallux valgus patients such as Lapidus fusion or first metatarsal osteotomy.

## Data Availability

No datasets were generated or analysed during the current study.

## Abbreviations

BMI: Body mass index
HV: Hallux valgus
1–2 IM angle: First-second intermetatarsal angle
ROC curve: Receiver operating characteristic curve
CI: Confident interval
Mm: Millimeter
ROC: Receiver Operating Characteristic
PPV: Positive predictive value
NPV: Negative predictive value
